# A cross-sectional population-based study on the influence of the COVID-19 pandemic on incomes in Greece

**DOI:** 10.3934/publichealth.2021029

**Published:** 2021-05-06

**Authors:** Dimitris Zavras

**Affiliations:** Department of Public Health Policy, School of Public Health, University of West Attica, Athens, Greece

**Keywords:** COVID-19, loss of income, social inequality, geographic region, tourism, employment status

## Abstract

The Coronavirus Disease 2019 (COVID-19) pandemic induced economic shock in Greece, which translated into a decrease in household income. Thus, the objective of this study is to measure social inequality with regard to income loss due to the COVID-19 pandemic in Greece. In addition, we aim to identify the characteristics of those experiencing income loss due to the pandemic. The study uses data from the “Public Opinion in the European Union (EU) in Time of Coronavirus Crisis. Third Round” survey. The sample consists of 1036 individuals aged between 16 and 54 years. To measure inequality, the Erreygers' Concentration Index (CI) is calculated, using social class as the ranking variable. To identify the characteristics of those experiencing income loss, a logistic regression model is fitted using the region of residence and several demographic and socioeconomic variables as potential predictors. According to the results, social inequality does not exist with regard to income loss due to the COVID-19 pandemic. Thus, our findings indicate the negative influence of the pandemic on the incomes of individuals from all social classes in Greece. According to the results of the logistic regression model, the odds of experiencing income loss are higher for residents of the Aegean Islands and Crete but also for self-employed, part-time employed, and unemployed individuals. These findings indicate the negative influence of the pandemic on Greek tourism and on sectors employing a large proportion of non-standard workers. Although inequality does not exist, a substantial proportion of those losing income due to the pandemic is in line with the global picture.

## 1. Introduction

In December 2019, an outbreak of pneumonia of unknown cause in Wuhan, Hubei province, China, raised attention not only within China but also internationally. By January 7, 2020, a novel coronavirus (CoV) had been isolated from patients in Wuhan [1]. On January 30, 2020, the World Health Organization (WHO) declared the Chinese outbreak of Coronavirus Disease 2019 (COVID-19) to be a public health emergency of international concern. Due to the rapid spread of COVID-19 from its origin to the rest of the world, on March 11, 2020, the WHO declared the COVID-19 outbreak to be a global pandemic. As of April 20, 2021, 141,754,944 confirmed cases and 3,025,835 deaths had been reported globally [2].

The COVID-19 pandemic has not only caused a medical crisis, but also resulted in an economic crisis [3], combining the characteristics of both supply shock and demand shock [4]. According to Kose and Sugawara, the global economy is facing a deep recession, the first of fourteen global recessions since 1870 to be triggered solely by a pandemic [5]. As generally is the case with economic crises, in which the security and stability of individuals' personal finances deteriorate [6], the COVID-19 pandemic is having tremendous socioeconomic impacts that include loss of household income through job loss or death of earning members, either temporary or permanent closure of small businesses, and a loss of businesses productivity [7]. Due to these negative effects, a large portion of society, mostly the more socioeconomically vulnerable, struggle to cope financially, physically, and mentally with the crisis [8]. Thus, socioeconomic vulnerability is of special importance due to its link with the risks of economic deprivation, as well as health risks [9]. Since the extent of a household's exposure to job and income losses during a recession is contingent on social class [10], the epidemic-induced economic shock affects individuals in different ways [11]. Specifically, lower socioeconomic groups are more likely to be more severely affected by the socioeconomic consequences of the COVID-19 pandemic [12].

In Greece, the first COVID-19 case was diagnosed on February 26, 2020, and the first death was reported on March 12, 2020. As of April 20, 2021, 316,879 confirmed cases and 9,540 deaths had been reported in Greece [2].

Some of the business and workplace suspensions implemented in Greece between March 10, 2020 and March 22, 2020, include a) the closure of schools and universities; b) a two-week closure of theatres, courthouses, cinemas, gyms, playgrounds, and clubs; c) the nationwide closure of shopping centers, cafes, restaurants, bars, museums, archaeological sites, and food outlets, excluding supermarkets and food outlets offering take-away and delivery only; d) the closure of all organized beaches and ski resorts; e) the closure of all hotels from March 22, 2020 midnight until the end of April; and f) the closure of all parks, recreation areas, and marinas. Further restrictions entered into force in April with regard to travel by land, sea, and air. Business restrictions were gradually relaxed by the end of May, but seasonal hotels only resumed operations on June 15. Until June 15, Athens' airport was the only one at which international flights were allowed; from June 15, international flights to Thessaloniki's airport resumed. On July 1, the ban on international flights from all countries except the United Kingdom and Sweden was lifted for all airports [13]. Entry to the country was allowed for travelers from the Schengen and associated countries, and from fifteen other countries. However, the USA, Russia and Turkey were not included among these countries, regardless of their significant contributions to Greece's gains from international tourism in previous years [14]. Further restrictions entered into force during the summer, including, among others, the closure of restaurants, bars, and clubs at midnight.

As a consequence of the measures implemented to reduce the spread of the coronavirus in Greece and most other countries, employment and disposable income were adversely affected [15]. However, data from the “Public Opinion in the European Union (EU) in Time of Coronavirus Crisis. Third Round” survey [16] indicate that not all counties were similarly affected by the pandemic. Indeed, regarding income loss due to the pandemic, Greece is ranked in the fourth highest place (38.32%) among the 27 EU member states; Luxemburg is ranked lowest (10.75%) and Hungary is the highest (41.65%). It is worth noting that approximately one-quarter (24.63%) of European citizens have experienced income loss due to the pandemic. It is to be expected that this percentage in Greece is higher than the average for Europe as a whole.

Nevertheless, the negative effects of the restrictions and flexible employment structures in Greece on income [17] do not automatically translate to socioeconomic inequality. That is, the vulnerability of lower socioeconomic status individuals regarding income loss as compared with higher socioeconomic status individuals should not be taken as a given [18]. As such, the objective of this study was to measure social inequality in terms of income loss due to the COVID-19 pandemic in Greece, but also to identify the characteristics of those experiencing this income loss.

## 2. Material and methods

For the purpose of this study, data from the “Public Opinion in the European Union (EU) in Time of Coronavirus Crisis. Third Round” [16] survey was used. A survey was conducted using Kantar's online access panel between September 25 and October 7, 2020 among 24,812 respondents in 27 EU Member States. The survey was limited to respondents aged between 16 and 64 years. In some countries, including Greece, the sample was limited to respondents aged between 16 and 54 years. Representativeness at the national level was ensured by quotas on gender, age, and region. The sample size was n = 1036 in Greece. Data collection took place between September 24 and September 27, 2020.

The respondents were asked the question, “Have you experienced any of the following, since the start of the coronavirus pandemic in your country?” “Loss of income” was among the financial issues under study. The potential answers were a) no (0) and b) yes (1). In addition, the respondents were asked the question, “Thinking about your personal income, which one of these statements comes closest to your current situation?” The potential answers were a) Coronavirus has already impacted on my personal impact; b) Coronavirus has not yet impacted on my personal income, but I expect it to in the future; and c) Coronavirus will have no impact on my personal income. The dichotomous variable “income loss due to the COVID-19 pandemic” was then derived from the combination of the abovementioned variables: a) no income loss (0) and b) income loss due to the COVID-19 pandemic (1).

To measure socioeconomic inequality as regards income loss due to the pandemic, a concentration index (CI), namely, Erreygers' CI, was calculated. CIs are used to measure inequality in one variable over the distribution of another [19]. The CI is defined with reference to the concentration curve, as twice the area between the curve and the 45° line of equality. The concentration curve plots the cumulative percentage of the variable under study (y-axis) against the cumulative percentage of the population, ranked by the socioeconomic variable, beginning with the least well-off, and ending with the most well-off (x-axis) [20]. The CI is given by:

$$CI=\frac{2}{nµ}\overset{n}{\underset{i=1}{\sum }}y_{i}R_{i}-1$$ (1)

where *n* is the number of individuals, *y* the variable under study, *µ* the mean of the variable under study, and *R_i_* the fractional rank. The minimum and maximum values of *CI* are −1 and 1, respectively [21].

In the case of bounded variables, such as the variable “income loss due to the COVID-19 pandemic”, the bounds of the CI depend upon the mean of the variable. In addition, the CI is not scale-invariant and does not satisfy the mirror property, on the basis of which the inequality indices of *y* and (1 − *y*) have equal absolute values but opposite signs. In these cases, the use of Erreygers' CI is recommended. Errreygers CI is given by:

$$Erreygers^{\prime }CI=\frac{4µ}{y^{max}-y^{min}}CI\;$$ (2)

with *y^max^* and *y^min^* as the bounds of the variable under study. Erreygers' CI lies in the interval [−1, 1]. Negative values indicate that the variable under study is concentrated among the least well-off respondents, whereas positive values indicate that the variable is concentrated among the most well-off [22], [23].

Social class was used as a ranking variable, as follows: a) low (1) semi-skilled or unskilled manual workers, students, retired and living on state pension only, unemployed (for over six months), or those not working due to long-term sickness; b) middle (2) skilled manual workers, supervisory or clerical/junior managerial/professional/administrator; and c) high (3) intermediate managerial/professional/administrative, higher managerial/professional/administrative. This variable was based on the occupation of the main earner of the household.

Because the response was binary, to identify the characteristics of those experiencing income loss due to the COVID-19 pandemic, a logistic regression model was fitted. As potential predictors in the analysis, the following variables were considered:

a) region: 1. Attica; 2. Macedonia and Thrace; 3. Epirus and Western Macedonia; 4. Thessaly and Central Greece; 5. Peloponnese, Western Greece and Ionian Islands; and 6. Aegean Islands and Crete.

b) gender: 0. female; and 1. male).

c) age.

d) marital status: 1. married/living with partner; 2. never married (single); 3. divorced/widowed; 4. living with parents; and 5. domestic partner/living with other adults.

e) household size: 1. 1; 2. 2; 3. 3; and 4. 4+.

f) social class: 1. low; 2. middle; and 3. high, as described above.

g) current employment status: 1. employed (full-time); 2. employed (part-time); 3. self-employed; 4. retired/unable to work/disabled; 5. still at school; 6. in full time higher education; 7. unemployed and seeking work; and 8. not working and not seeking work.

Helmert coding was applied to the ordinal variables: a) social class; and b) household size. The Helmert contrast compares each category of an ordinal variable (except the last) with the differences from the balanced mean of the subsequent levels. Indicator coding was applied to the nominal variables: a) region; b) marital status; and c) current employment status. Indicator contrast was used to compare the reference category of a nominal variable with the remaining categories. The binary variable “gender”, was treated as such.

The model's goodness of fit was tested through the Hosmer and Lemeshow test. The calibration of the model was tested through the calibration belt test. In addition, the model was tested for specification error through the link test.

The STATA 14 statistical software package was used for the analysis. Specifically, the commands conindex [19] desmat [24], logistic, linktest, and calibrationbelt [25], and were used.

## 3. Results

As mentioned in the material and methods section, data collection took place online between September 24 and September 27, 2020.

Regarding gender, 50.68% of the respondents were female and 49.32% were male. The mean age of the respondents was 37.34 years (±10.28), while 16.22% of the respondents were aged between 16 and 24 years, 22.39% were between 25 and 34 years, 30.21% were between 35 and 44 years, and 31.18% were between 45 and 54 years. The characteristics of the respondents are presented in Table 1.

**Table 1.** Characteristics of the respondents. Dates of interviews: 9/24–9/27, 2020.

Geographic Region	Gender-Age: Male % (n)				Gender-Age: Female % (n)			
	16–24	25–34	35–44	45–54	16–24	25–34	35–44	45–54
Attica	2.80 (29)	4.15 (43)	3.96 (41)	5.98 (62)	3.09 (32)	4.44 (46)	4.54 (47)	7.43 (77)
Macedonia and Thrace	1.54 (16)	2.51 (26)	4.63 (48)	4.44 (46)	2.12 (22)	1.93 (20)	2.99 (31)	3.47 (36)
Epirus and Western Macedonia	0.39 (4)	0.19 (2)	1.06 (11)	1.16 (12)	0.29 (3)	0.58 (6)	1.35 (14)	0.87 (9)
Thessaly and Central Greece	0.87 (9)	1.35 (14)	1.93 (20)	1.25 (13)	1.16 (12)	1.25 (13)	2.32 (24)	1.45 (15)
Peloponnese, Western Greece and Ionian Islands	1.16 (12)	1.54 (16)	1.64 (17)	1.64 (17)	1.16 (12)	2.22 (23)	2.32 (24)	1.16 (12)
Aegean Islands and Crete	0.87 (9)	1.25 (13)	1.83 (19)	1.16 (12)	0.77 (8)	0.97 (10)	1.64 (17)	1.16 (12)

According to the descriptive analysis, 38.32% of the respondents experienced income loss due to the COVID-19 pandemic.

Erreygers' CI for experiencing income loss due to the COVID-19 pandemic was found to be equal to −0.039 (p = 0.251). According to this finding, the Erreygers' CI value was not significantly different from zero, indicating a lack of social inequality.

According to the logistic regression model, the odds of experiencing income loss due to the COVID-19 pandemic are higher for residents of Aegean Islands and Crete and for part-time employed, self-employed, and unemployed individuals who are seeking work (Table 2).

**Table 2.** Logistic regression model. Dates of interviews: 9/24–9/27, 2020; Gender: Male-Female; Age: 16–54.

Variable	Odds Ratio	p	95% Confidence Interval	
Region		0.011		
Macedonia and Thrace	1.101	0.599	0.770	1.573
Epirus and Western Macedonia	0.677	0.221	0.363	1.265
Thessaly and Central Greece	0.705	0.150	0.439	1.134
Peloponnese, Western Greece and Ionian Islands	0.885	0.591	0.566	1.383
Aegean Islands and Crete	2.002	0.005	1.227	3.268
Current Employment Status		<0.001		
Employed (Part-Time: Less than 30 Hours per Week)	2.346	<0.001	1.535	3.587
Self-Employed	4.617	<0.001	2.816	7.570
Retired/Unable to Work/Disable	0.451	0.311	0.097	2.103
Still at School	6.549	0.109	0.656	65.393
In Full-Time Higher Education	0.866	0.647	0.469	1.601
Unemployed and Seeking Work	1.751	0.002	1.231	2.489
Not Working and not Seeking Work	0.976	0.962	0.364	2.620
Constant	0.425	<0.001	0.326	0.554

According to the link test (Table 3), the model does not suffer from specification error.

**Table 3.** Link test. Dates of interviews: 9/24–9/27, 2020; Gender: Male-Female; Age: 16–54.

Variable	Coefficient	p	95% Confidence Interval	
h	1.021	<0.001	0.718	1.323
h^2^	0.040	0.818	–0.298	0.378
Constant	–0.011	0.912	–0.213	0.190

The Hosmer and Lemeshow test indicated a good fit (p = 0.869). In addition, the calibration belt test indicated good calibration (p = 0.817).

## 4. Conclusions

The world economy and financial markets have been severely impacted by the COVID-19 pandemic [26]. The pandemic and the mitigation policies implemented have had immediate labor market consequences, such as an exponential increase in unemployment [27]. Thus, the epidemic-induced economic shock has been translated into an immediate fall in household incomes [28].

In general, the economic consequences of the pandemic have disproportionally impacted individuals or households of different socioeconomic statuses [29]. Specifically, the economic impact of the pandemic has been higher for those in the lowest socioeconomic strata [30]; that is, financial pressure has been higher for vulnerable socioeconomic groups [31]. In other terms, the COVID-19 pandemic through a) its unequal health burden and b) its disparity of economic losses has set marginalized groups of the society up to be more vulnerable [32].

However, evidence from Brazil indicates that all social classes have suffered from income reduction due to the lockdown [33]. In addition, evidence from the USA indicates that approximately one-third of households have experienced an income reduction, regardless of whether their household income before the COVID-19 crisis was less than $25,000 per year, $25,000 to $124,999 per year, or $125,000 and more per year [34]. That is, the COVID-19 pandemic has induced economic chaos which has affected all social classes [35].

Thus, although the results of our study regarding the non-existence of inequality are consistent with a limited strand of literature, they confirm concerns that the COVID-19 pandemic is expected to have severe negative effects on almost all sectors of economic activity in Greece [36]. Fears of income loss were confirmed as a result of the implemented restrictions and the demand shock that affected most sectors of the economy, causing a major economic slowdown that significantly impacted businesses and workers [37]. In this sense, the well-documented association between the economic sector in which one works and the risk of income loss [38] justifies our results.

According to the logistic regression model, the odds of experiencing income loss are higher for residents of Aegean Islands and Crete as compared with residents of Attica. The most probable reason for this is the supply and demand disruptions due to the high degree of dependence of these regions on tourism. Indeed, Greek tourism has been severely hit by the COVID-19 pandemic [39].

The findings with regard to current employment status reflect the fact that the economic sectors most directly affected by the COVID-19 containment measures in Greece a) employ a large proportion of non-standard workers (i.e., part-time workers, self-employed workers, and workers hired on fixed-term contracts) and b) these sectors account for more than 50% of total employment [40]. These findings confirm data from the literature showing that the most workers who are most vulnerable to income loss are the self-employed and individual entrepreneurs, the workers of the industries most affected by the coronavirus, and the workers whose employment status was precarious before the pandemic [41].

It is evident that hundreds of millions of individuals globally have been exposed to severe financial uncertainty by the COVID-19 pandemic [42] through job and income losses [43] that have increased both job insecurity [44] and financial insecurity [45].

The importance of studying the influence of the pandemic on incomes relates to the well-documented relationship between income, health and well-being; in the case of the COVID-19 pandemic, its two interlinked aspects, i.e., the health crisis and the economic crisis, put people at the double risk of health deterioration and loss of income [36]. The reasons for why individual income matters for health includes its link with material deprivation as well as its link with restrictions on social participation and opportunity to exercise control over one's life [46]. In other terms, “economic and social circumstances affect health through the physiological effects of their emotional and social meanings and the direct effects of material circumstances” [47]. That is, income provides the means to acquire necessities for life, gain access to health-enhancing resources, avoid harmful exposures, and participate in normal social activities, while a low income is linked with psychosocial stress [48]. Thus, losing income is not only linked with the inability to cope with daily survival costs [49], but it is also linked with exposure to health and safety risks, such as homelessness or food insecurity [50]. Therefore, because individuals and households are facing most of the challenges mentioned above due to the COVID-19 pandemic [51], income support measures have been implemented in many countries including Greece.

Research findings focusing on the economic crisis of 2008 in Greece provide evidence of its negative effect on factors including living and welfare standards [52], financial access to healthcare [53], and health [54], through the considerable income cuts that have occurred. Considering that the economic impact of the COVID-19 pandemic will be more severe than that of the 2008 crisis [39], we may argue that income loss in Greece during the current pandemic constitutes a threat for the health and well-being of the Greek people.

Greece has experienced a massive and prolonged economic recession, and the COVID-19 pandemic is slowing down its recovery efforts. Although the country's economy faces a high level of uncertainty, the Greek government has implemented a broad range of measures to support households, businesses, and the economy overall in this difficult time [55]. Income support measures (i.e., state income benefits to support freelancers, self-employed people, and sole proprietors), financial support for suspended and dismissed employees, support for seasonal employees, and financial support for seasonal employees not re-hired in 2020 were applied nationwide [56]. Those employed in the tourism industry, which has been severely affected by the pandemic, were eligible to receive support in the amount of 534 € monthly if their employment contracts were suspended. To also receive the extraordinary monthly support (equal to the last unemployment allowance) from June to August 2020, seasonal tourism employees were required to: a) be employed either full- or part-time in 2019 and b) receive unemployment allowances for three months and five days from September 2019 to February 2020. To be eligible for unemployment allowances in 2020, seasonal workers in the tourism and restaurant industries were required to collect 50 social security stamps instead of 100, the previous requirement [57]. On 20 October 2020, the European Commission also approved a 450 million € Greek scheme to support tourism and transport companies, among others [58]. Furthermore, because firm size and geographic location were among the primary drivers of exposure to the COVID-19 shock [59], on 28 September 2020, the European Commission approved 1.5 billion € for 12 impacted Greek regions to support micro and small enterprises as well as those based on islands. Crete and the Aegean Islands were included. Enterprises facing sudden liquidity shortages will receive direct grants for working capital; the amount will equal a percentage of the expenses that the beneficiaries incurred in 2019, up to 50% [58].

However, to minimize job losses and business closures in the short and medium terms, the Greek government must take coordinated action through policy at the local, regional and national levels [60]. Nevertheless, since the economic impact of the COVID-19 pandemic differs across regions, as the results of this study confirm, tailored governance and policy responses are required [61]. Of course, such responses should take into consideration socioeconomic vulnerability, demographic vulnerability, vulnerability due to housing and hygiene conditions, vulnerability due to non-availability of healthcare and epidemiological vulnerability [62].

## References

[1] Wang C, Horby PW, Hayden FG (2020). A novel coronavirus outbreak of global health concern. Lancet.

[2] WHO WHO coronavirus disease (COVID-19) dashboard, 2021.

[3] Susskind D, Vines D (2020). The economics of the COVID-19 pandemic: an assessment. Oxf Rev Econ Policy.

[4] Furman J, Baldwin RE, Weder B (2020). Protecting people now, helping the economy rebound later. Mitigating the COVID economic crisis act fast and do whatever it takes.

[5] World Bank (2020). Global economic prospects, June 2020.

[6] Marjanovic Z, Greenglass ER, Fiksenbaum L (2013). Psychometric evaluation of the Financial Threat Scale (FTS) in the context of the great recession. J Econ Psychol.

[7] Baddour K, Kudrick LD, Neopaney A (2020). Potential impact of the COVID-19 pandemic on financial toxicity in cancer survivors. Head Neck.

[8] The Lancet (2020). Redefining vulnerability in the era of COVID-19. Lancet.

[9] Finch A, Tribble AG (2021). The path ahead: From global pandemic to health promotion. Prev Med Rep.

[10] Witteveen D (2020). Sociodemographic inequality in exposure to COVID-19-Induced economic hardship in the United Kingdom. Res Soc Stratification Mob.

[11] Blundell R, Costa Dias M, Joyce R (2020). COVID-19 and inequalities. Fisc Stud.

[12] Burström B, Tao W (2020). Social determinants of health and inequalities in COVID-19. Eur J Public Health.

[13] Economou C, Kaitelidou D, Konstantakopoulou O (2020). Preventing transmission. Policy responses: Greece.

[14] Foundation for Economic & Industrial Research (2020). The Greek economy. Quarterly Bulletin VOL. 2/20.

[15] Marsellou E, Liargovas PG (2020). Consumer price index fell during COVID-19 lockdown. Greek economic outlook.

[16] European Parliament (2020). Public opinion in the EU in time of coronavirus crisis. Third round.

[17] Hazakis KJ (2021). Is there a way out of the crisis? Macroeconomic challenges for Greece after the Covid-19 pandemic. Eur Polit Soc.

[18] Gaviria A (2002). Household responses to adverse income shocks in Latin America. Revista Dessarolo y Sociedad.

[19] O'Donnell O, O'Neill S, Van Ourti T (2016). Conindex: estimation of concentration indices. Stata J.

[20] O'Donnell O, van Doorslaer E, Wagstaff A (2007). Analyzing health equity using household survey data: A guide to techniques and their implementation. World Bank.

[21] Kakwani N, Wagstaff A, van Doorslaer E (1997). Socioeconomic inqualities in health: Measurement, computation, and statistical inference. J Econom.

[22] Erreygers G (2009). Correcting the concentration index. J Health Econ.

[23] van Doorslaer E, Van Ourti T, Glied S, Smith PC (2013). Measuring inequality and inequity in health and health care. The Oxford handbook of health economics.

[24] Hendrickx J (1999). Stata technical Bulletin-52, using categorical variables in Stata.

[25] Nattino G, Lemeshow S, Phillips G (2017). Assessing the calibration of dichotomous outcome models with the calibration belt. Stata J.

[26] Park CL, Russell BS, Fendrich M (2020). Americans' COVID-19 Stress, Coping, and Adherence to CDC Guidelines. J Gen Intern Med.

[27] Settersten RA, Bernardi L, Härkönen J (2020). Understanding the effects of Covid-19 through a life course lens. Adv Life Course Res.

[28] Sarkar P, Debnath N, Reang D (2021). Coupled human-environment system amid COVID-19 crisis: A conceptual model to understand the nexus. Sci Total Environ.

[29] Kansiime MK, Tambo JA, Mugambi I (2021). COVID-19 implications on household income and food security in Kenya and Uganda: findings from a rapid assessment. World Dev.

[30] Zaidel EJ, Forsyth CJ, Novick G (2020). COVID-19: implications for people with Chagas disease. Glob Heart.

[31] Levine DT, Morton J, O'Reilly M (2020). Child safety, protection, and safeguarding in the time of COVID-19 in Great Britain: proposing a conceptual framework. Child Abuse Negl.

[32] Ali S, Asaria M, Stranges S (2020). COVID-19 and inequality: are we all in this together?. Can J Public Health.

[33] Leone T (2020). COVID-19 Sends the Bill: Socially disadvantaged workers suffer the severest losses in earnings. Latin American and the Caribbean economic association working papers Seres. No 0050.

[34] Carman K, Nataraj S (2020). How are Americans paying their bills during the COVID-19 pandemic?. Rand Corporation.

[35] Sampaio FJB (2020). Reflections on the COVID-19 pandemic. Int Braz J Urol.

[36] Petrakis PE, Kostis PC (2020). The Evolution of the Greek Economy: Past Challenges and Future Approaches.

[37] Betcherman G, Giannakopoulos N, Laliotis I (2020). Reacting quickly and protecting jobs. The short-term impacts of the COVID-19 lockdown on the Greek labor market. World Bank Group.

[38] Finseraas H, Ringdal K, Ervasti H, Andersen JG, Fridberg T (2012). Economic globalization, personal risks and the demand for a comprehensive welfare state. The future of the welfare state.

[39] Papanikos GT (2020). The impact of the Covid-19 pandemic on Greek tourism. Athens J Tourism.

[40] Organization for Economic Co-operation and Development (2020). Distributional risks associated with non-standard work: Stylised facts and policy considerations.

[41] Kartseva MA, Kuznetsova PO (2020). The economic consequences of the coronavirus pandemic: which groups will suffer more in terms of loss of employment and income?. Popul Econ.

[42] Hansel TC, Saltzman LY, Bordnick PS (2020). Behavioral health and response for COVID-19. Disaster Med Public Health Prep.

[43] Foster G (2020). Early estimates of the impact of COVID-19 disruptions on jobs, wages, and lifetime earnings of schoolchildren in Australia. Aust J Lab Econ.

[44] Armitage R, Nellums LB (2020). COVID-19: compounding the health-related harms of human trafficking. Clin Med.

[45] Brener A, Mazor-Aronovitch K, Rachmiel M (2020). Lessons Learned from the Continuous Glucose Monitoring Metrics in Pediatric Patients with Type 1 Diabetes under COVID-19 Lockdown. Acta Diabetologica.

[46] Marmot M (2002). The influence of income on health: views of an epidemiologist. Health Aff.

[47] Marmot M, Wilkinson RG (2001). Psychosocial and material pathways in the relation between income and health: A response to Lynch et al. BMJ.

[48] Douglas M, Katikireddi SV, Taulbut M (2020). Mitigating the wider health effects of Covid-19 pandemic response. BMJ.

[49] Kajdy A, Feduniw S, Ajdacka U (2020). Risk factors for anxiety and depression among pregnant women during the COVID-19 pandemic: A web-based cross-sectional survey. Medicine.

[50] Jay J, Bor J, Nsoesie EO (2020). Neighbourhood income and physical distancing during the COVID-19 pandemic in the United States. Nat Hum Behav.

[51] Glover RE, van Schalkwyk MCI, Akl EA (2020). A framework for identifying and mitigating the equity harms of COVID-19 policy interventions. J Clin Epidemiol.

[52] Papanastasiou S, Papatheodorou C (2018). The Greek depression: poverty outcomes and welfare responses. East West J Econ Bus.

[53] Zavras D (2020). Studying healthcare affordability during an economic recession: the case of Greece. Int J Environ Res Public Health.

[54] Chantzaras A, Yfantopoulos J (2017). The effects of the economic crisis on health status and health inequalities in Greece. Value Health.

[55] Adrikopoulou C (2020). Greece COVID-19. Eur State Aid Law Q.

[56] European Foundation for the Improvement of Living and Working Conditions (2020). COVID-19 EU policywatch. Database of national-level responses.

[57] European Systemic Risk Board (2021). Greece. Measures taken in response to coronavirus (COVID-19) pandemic.

[58] European Commission (2021). Greece. Details of Greece's support measures to help citizens and companies during the significant economic impact of the coronavirus pandemic.

[59] International Monetary Fund (2020). Greece. IMF country report No.20/308.

[60] Organisation for Economic Co-operation and Development (2020). Regional policy for Greece post-2020. OECD Territorial Reviews.

[61] Organisation for Economic Co-operation and Development (2020). The territorial impact of COVID-19: Managing the crisis across levels of government.

[62] Acharya R, Porwal A (2020). A vulnerability index for the management of and response to the COVID-19 epidemic in India: An ecological study. Lancet Glob Health.

